# Insight into Oil-in-Water Emulsions Stabilized by Cross-Linked and Pregelatinized Starches: The Effect of Molecular Structure, Surface Activity and Proton Molecular Dynamics

**DOI:** 10.3390/molecules29235626

**Published:** 2024-11-28

**Authors:** Joanna Le Thanh-Blicharz, Jacek Lewandowicz, Artur Szwengiel, Krystyna Prochaska, Hanna Maria Baranowska, Grażyna Lewandowicz

**Affiliations:** 1 Department of Food Concentrates and Starch Products, Prof. Wacław Dąbrowski Institute of Agriculture and Food Biotechnology—State Research Institute, Starołęcka 40, 61-361 Poznań, Poland; joanna.lethanh-blicharz@ibprs.pl (J.L.T.-B.); jacek.lewandowicz@ibprs.pl (J.L.); 2 Department of Food Technology of Plant Origin, Faculty of Food Science and Nutrition, Poznan University of Life Sciences, Wojska Polskiego 31, 60-624 Poznań, Poland; artur.szwengiel@up.poznan.pl; 3 Institute of Chemical Technology and Engineering, Poznań University of Technology, Berdychowo 4, 60-965 Poznan, Poland; krystyna.prochaska@put.poznan.pl; 4 Department of Physics and Biophysics, Faculty of Food Science and Nutrition, Poznań University of Life Sciences, Wojska Polskiego 38/42, 60-637 Poznań, Poland; hanna.baranowska@up.poznan.pl; 5 Department of Biotechnology and Food Microbiology, Faculty of Food Science and Nutrition, Poznań University of Life Sciences, Wojska Polskiego 48, 60-627 Poznań, Poland

**Keywords:** starch modification, emulsion stability, low field nuclear magnetic resonance, size exclusion chromatography, surface/interfacial tensions, rheological properties

## Abstract

Effective formation and stabilisation of emulsions while meeting high consumer requirements, including the so-called green label, is still a technological challenge. This is related to the multitude of emulsion destabilization mechanisms and the vastness of methods used to study them, which implies the need to develop an understanding of the phenomena occurring in emulsions. Commercial starch preparations obtained by physical and chemical modification were used to prepare model emulsions that were studied in terms of their stability. Native potato starch was the reference material. The analytical methods used included rheology, low field nuclear magnetic resonance (LF NMR), size exclusion chromatography with triple detection (SEC), and surface/interfacial tension measurements. The results showed that chemical and physical modification improved the functionality of starch in emulsions. This is due to not only chemical but also physical modifications, i.e., pregelatinization causes changes in the molecular structure of starch, including an increase in the molecular weight and the degree of branching. As a consequence, the conformation of starch macromolecules changes, which results in a change of the dynamics of protons in the continuous phase of the emulsion and the thermodynamics of starch adsorption at the water/oil interface.

## 1. Introduction

Many food products, such as mayonnaises, sauces, dips, creams, spreads, and pâtés, constitute emulsion systems. They are thermodynamically unstable, so their efficient production and stabilization is still one of the most challenging issues for food technologists. The instability of emulsions results from the fact that they are a colloidal dispersion of two immiscible liquids and their formation requires energy to increase the interfacial surface. Hence, the basic technological strategy is the introduction of surfactants, i.e., substances able to reduce the interfacial tension. Food grade surfactants are called emulsifiers. However, emulsion breakdown can occur by many different mechanisms such as coalescence, flocculation, gravitational separation or phase separation. Therefore, not only emulsifiers that primarily enable short-term stability of the emulsions, but also the so-called stabilizers are used to improve the long-term stability of these systems. Various hydrocolloids, such as proteins and polysaccharides, especially starches, both in their native and modified form, are most often used in this task. Moreover, in relation to hydrocolloids there is no clear distinction between the terms emulsifier and stabilizer, and often they are used alternatively [1,2,3,4,5,6,7,8]. The increasing interest in particle-stabilized emulsions called Pickering emulsions has been observed in recent years. Traditional and Pickering emulsions differ fundamentally in the physicochemical properties of substances present at the oil–water interface. While traditional emulsions are produced, thanks to adsorption at the oil/water interface of the soluble, amphiphilic molecules of emulsifiers, Pickering emulsions are stabilized by the presence of insoluble, submicron particles at the oil–water interface. Some advantages of Pickering emulsions over traditional emulsions have been mentioned in the scientific literature, but in industrial practice, the latter still dominates. Moreover, a lot of theoretical and practical issues regarding traditional emulsions remain unsolved [9,10,11,12].

A multiplicity of mechanisms of destabilization of emulsions implies plurality in analytical methods for their study. The most popular are; microscopic methods; particle size measurements; and texture measurements, especially those employing rheological methods; and accelerated stability tests, mainly via centrifugation and fats oxidation analyses. Excellent, comprehensive reviews of the techniques and methodologies in this regard can be found in papers by McClements [13] and Kupikowska-Stobba et al. [14]. A less known, but very beneficial method for studying emulsions is low-field nuclear magnetic resonance (LF NMR). It is designed to study the dynamics of protons, so any system, even very complex that is rich in hydrogen, can be analysed by employing this technique. Hence, it has been used in the studies of different biological systems (including food) in their native form and environment. Most often, the proton dynamics of water molecules are studied as water is abundant in biological materials. Relaxation phenomena of protons that interact with an external magnetic field are observed. The spin-lattice relaxation time T_1_, corresponding to the transfer of spin energy to the environment, and the spin-spin relaxation time T_2_, corresponding to the mutual transfer of spin energy, are measured. The value of the spin-lattice relaxation time is influenced by many factors, including temperature, resonance frequency or the presence of macromolecules. The T_1_ value of water increases linearly with increasing field intensity, whereas it significantly decreases as a result of the dissolution of proteins or polysaccharides [15,16,17,18]. Not only the molecular dynamics of water are studied using LF NMR but also phenomena occurring in fats, in spite of the fact that the values of relaxation times in fats are much lower than in aqueous systems. Moreover, special attention should be paid to the effect of the formation of emulsion systems on the proton relaxation phenomena. The formation of an emulsion, i.e., a colloidal dispersed system, complicates the proton relaxation processes. Two components of each relaxation time are observed, which proves that there are two proton fractions in the system, relaxing at different rates [19].

Starch is the most important source of energy in the human diet. However, its technological importance is often identified as its ability to shape the texture of food. Therefore, a lot of effort was made to control the rheological properties of starch [20,21,22,23,24,25,26]. As a result, both traditional physical, chemical and enzymatic processes as well as several less popular innovative methods for starch modification that change its structure are employed. This results in a change in the physicochemical and functional properties of this polysaccharide [8,27,28,29]. This also makes it possible to control its surface activity. Chemically modified starches can decrease interfacial tension at both air/water and oil/water interfaces to a higher extent than native starch. The effect obtained depends on both the type of modification and the degree of substitution. Further increases in the ability to reduce surface and interfacial tension can be achieved by enzymatic hydrolysis of starch preparations [30,31].

Food-grade cross-linked starches stand out among the chemically modified starches by their very low degree of substitution, an order of magnitude lower than those of food mono-starch derivatives. For example, in cases of acetylation, maximally one in ten anhydroglucose units of starch macromolecules can contain the modifying acetyl groups;

– acetylated starch: starch–O–CO–O–CH_3_,
  whereas cross-linked derivatives can be substituted at most only in one in five hundred anhydroglucose molecules [32,33];
– distarch phosphate: starch–O–PO_2_–O–starch;
– distarch adipate: starch–O–CO–(CH_2_)_4_–CO–O–starch.

Nevertheless, they provide excellent technological properties thanks to an increase in the molar mass of starch that increases the viscosity of pastes, and their resistance to extended cooking times, acidity or shear stress. Distarch phosphates E1412, prevent leakage by heat treatment of meat products so they are often used in the production of ham or sausages. Double-modified starches: acetylated distarch phosphate E1414 and acetylated distarch adipate, which contain slightly hydrophobic acetyl groups and reveal higher surface activity than native starch, are often used in the manufacture of mayonnaises, sauces, margarines, or different reduced-fat products [20,34,35].

Despite chemically modified starches providing much better results, both in terms of technology and economy, consumers lately prefer foods with so-called clean labels, which in the case of starch means restrictions in the use of chemically modified ones [36,37]. Therefore, an increase in the significance of physical modification methods is observed. Pregelatinization is the most common process for physical modification of starch, despite the small decrease in the paste’s viscosity. Pregelatinization in industrial practice is carried out by drying a starch suspension by employing drum dryers. It provides starch the ability to swell in cold water and create a colloidal solution due to the amorphization of its granules [38,39,40]. Physical modification of starch generally means the action of physical factors, such as heat, moisture, shear, radiation or pressure, without involving any chemicals. However, the production of granular cold water soluble (GCWS) starches is recognized as a physical modification despite involving heating of starches in aqueous alcohol and alcoholic alkaline treatment. This is of special importance concerning emulsion technology as pregelatinized and GCWS starches increase emulsion stability [6,35,41,42]. Other physical modification methods, including among others high-pressure homogenization of pastes, are also important for increasing the functionality of starch in emulsion systems [43]. The information presented above points to the need for further studies on the mechanisms of emulsion stabilization by starches modified by various methods. Cross-linked starches are commercially available not only in granular form but also as pregelatinized ones. Although they are a little bit more expensive, the latter is often chosen by food manufacturers. This may be related to the simplification of the technological process, but it is also suggested these starches have better functionality of pregelatinization in some applications. Therefore, this study aimed to find out the effect of sole chemical or physical as well as dual chemical-physical modification of starch on the structure, rheological properties, surface activity and functionality in emulsions of commercially available preparations of E1412, E1414 and E1422.

## 2. Results and Discussion

### 2.1. Stability of Formed Emulsions

**Figure 1:**
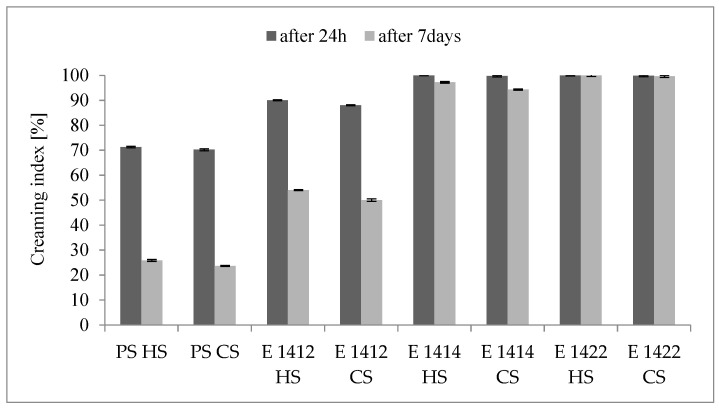
Changes in the creaming index of the emulsions during storage (PS—potato starch; HS—starches soluble in hot water; CS—starches soluble in cold water).

As described in the introduction, emulsion stability is a complex concept and a lot of methods could be applied to analyse it. Nevertheless, the traditional, simple method employing the centrifugation process provides useful results and is very common. Very diverse values of creaming index data have been provided in the literature, which is related to the huge variety of emulsion recipes and methods for their production [44,45,46,47,48]. Nevertheless, the creaming index values are perfect for comparing different emulsifiers, stabilizers or conditions for emulsion preparation within a particular experiment. As it is presented in Figure 1, cross-linked starches revealed much better emulsifying properties than chemically unmodified starch. This applies to both cold-water and hot-water soluble preparations. The creaming index decreased significantly after 24 h, but in cross-linked and additionally acetylated starches this decrease was only slight.

Another way to study the stability of the emulsions is to analyse colour parameters as light scattering depends on the composition and microstructure of colloidal dispersed systems [49,50,51]. The data presented in Table 1 indicate that chemically unmodified starch led to the production of the lowest lightness parameters (L). As in the case of the creaming index, better results were obtained for cross-linked starches, especially if they were additionally subjected to acetylation. It is also worth noting that in all cases, lighter emulsions were obtained using cold-water soluble preparations (CS) than their hot-water soluble analogues (HS). This is following the observations reported in the literature that pregelatinized starches are particularly useful in emulsion preparation [42]. Changes in lightness were accompanied by changes in parameters chromatic a* and b*. Lighter emulsions were characterized by more intense green (a) and yellow (b) colours. In the case of emulsions based on CS preparations, the shift towards the yellow colour was smaller. The lightest emulsion was obtained employing acetylated distarch adipate. Emulsions containing acetylated distarch phosphate differed in colour only slightly. More significant differences were observed for both the emulsions based on chemically unmodified starch and distarch phosphate.

### 2.2. Rheological Properties of Emulsions

The measurement of the rheological properties of emulsions is of considerable practical importance as many of their sensory features correspond to specific parameters measured instrumentally. The rheological properties of an emulsion depend on many factors, the most important of which are the viscosity of the continuous phase and the concentration and structure of the dispersed phase [1,4,52]. The parameters of the Ostwald de Waele equation, which describe the flow curves of the studied emulsions, are presented in Table 2. The applied model fits the analytical data very well (R ≥ 0.98). Among the presented data, the most noteworthy are clear differences between the rheological properties of emulsions based on cold and hot soluble starches. The values of the consistency index, which correspond to the sensory perception of the product’s viscosity, in the case of emulsions with HS preparations increased as a result of the use of cross-linked starches. This particularly concerned distarch phosphate E1412, not submitted to an additional acetylation process. These increases in the consistency index were accompanied by a decrease in the flow behaviour index. In contrast, emulsions prepared with CS preparations revealed significantly smaller differences in these rheological parameters. This points to an essential effect of physical modification on the functional properties of the starches studied. As regards thixotropy, which describes the rheological instability of the fluids, it was observed that its lower values positively correlate with a higher creaming index of the emulsion. The high thixotropy values revealed emulsions based on PS CS and E1412 CS preparations. However, this phenomenon was significantly reduced in the case of starch preparations subjected to the acetylation process.

The viscosity of fluids is largely dependent on the temperature. This phenomenon is related to the energy that is supplied to the sample, which changes the interactions and intermolecular friction during flow [53,54,55]. The analysed cold-soluble starches created emulsions that revealed higher values of flow activation energy than their hot-soluble counterparts. A particularly large difference in activation energy values occurred in the case of E1412.

**Figure 2:**
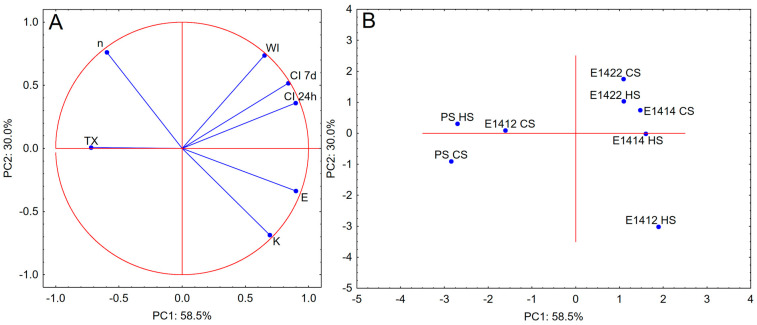
PCA analysis—(**A**) (loadings plot) and (**B**) (score plot) for parameters of stability of the emulsions and their rheological properties (CI 24 h—creaming index after 24 h of storage; CI 7 d—creaming index after 7 days of storage; WI—whiteness index; K—consistency index; n—flow behaviour index; E—flow activation energy; TX—thixotropy; PS HS—native potato starch; PS CS pregelatinized potato starch; E1412 HS—distarch phosphate; E1412 CS—pregelatinized distarch phosphate; E1414 HS acetylated distarch phosphate; E1414 CS—pregelatinized acetylated distarch phosphate; E1422 HS acetylated distarch adipate; E1414 CS—pregelatinized acetylated distarch adipate).

PCA analysis including the data of creaming index and lightness as the parameters describing stability of emulsions as well as rheological data is presented in Figure 2. The presented PCA plots represent over 88% of the total variation between samples. Acetylation was found important for controlling the functionality of starches in the control of emulsion rheology as four preparations of cross-linked and acetylated starches formed one group on the PCA score plot. Both preparations of chemically unmodified starch and cross-linked pregelatinized starch were also found to be similar. The most different from the other preparations was distarch phosphate HS. However, the location of vectors indicates a weak correlation of the parameters considered. The highest, albeit negative, correlation was observed for thixotropy, which is in its essence a measure of the rheological instability of the system. A slight positive correlation was observed between the creaming index and activation energy values.

### 2.3. Molecular Dynamics of Emulsions

Two components of the T_1_ and T_2_ relaxation times were observed in the emulsion studied (Table 3). This means that there are two fractions of protons in the system that relax at different rates and that the chemical exchange between these fractions of protons is much slower than the relaxation time. Long components (T_12_ and T_22_) reflect the relaxation processes of protons contained in the starch paste, while short components (T_11_ and T_21_) refer to the fat fraction of the emulsion. In the emulsions formed, the values of short components were significantly lower than the values of relaxation times as in pure oil T_1_ = 102.8 ± 0.8 ms and T_2_ = 88.4 ± 1.1 ms were measured. The shortening of relaxation times depended on the type of starch and usually decreased further during storage for 24 h except emulsions based on chemically unmodified starch. An especially significant increase, in the case of the T_11_ relaxation time, was observed. That indicates some instability of the emulsion. Cross-linked starches revealed higher relaxation times; however, after 24 h, the T_11_ and T_12_ values slightly decreased. The exception are emulsions based on acetylated distarch adipate, for which no changes in the short components of relaxation times were observed. Additionally, it was found that the fat fraction of emulsions obtained from HS and CS had almost no differences. A strong decrease in the relaxation times was also observed for the continuous phase of the emulsion, as for pure water the values T_1_ = 2760 ms and T_2_ = 2500 ms were measured. The long components T_12_ and T_22_ of relaxation times differed due to the modification method applied (Table 3). Primarily, it is worth noting that all CS preparations formed emulsions for which the relaxation times T_12_ and T_22_ nearly did not change over time. This means that starches soluble in cold water formed emulsions that are more stable in terms of proton relaxation phenomena in the water phase.

**Figure 3:**
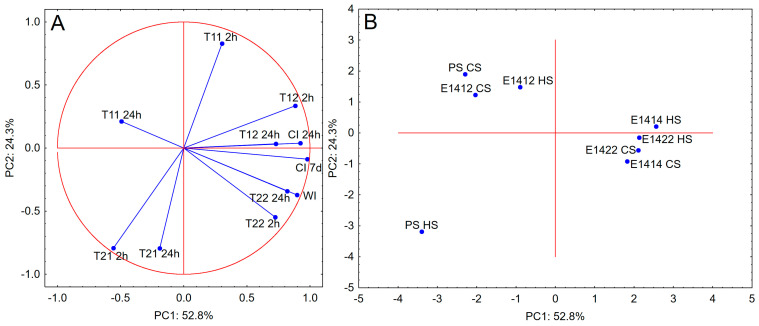
PCA analysis—(**A**) (loadings plot) and (**B**) (score plot) for parameters of stability of the emulsions and the parameters of proton relaxation phenomena (CI 24 h—creaming index after 24 h of storage; CI 7 d—creaming index after 7 days of storage; WI—whiteness index; T_11_—short component of the spin-lattice relaxation time (measured after 2 h or 24 h); T_21_—short component of the spin-spin relaxation time (measured after 2 h or 24 h); T_12_—long component of the spin-lattice relaxation time (measured after 2 h or 24 h); T_22_—long component of the spin-spin relaxation time (measured after 2 h or 24 h).

PCA analysis including the data describing the stability of emulsions and proton relaxation times (Figure 3), in contrast to rheological properties, proves a good correlation between the analysed parameters. A positive correlation was observed between the emulsion stability parameters and the relaxation times T_22_ and T_12_. A high negative correlation was observed for T_11_ 24 h. The relaxation times T_21_ and T_11_ 2 h did not show any significant correlation with the creaming and whiteness indexes. The presented PCA plots represent 77% of the total variation between samples. All cross-linked and acetylated starches were very close to each other. Both samples of distarch phosphate were close to samples subjected only to physical modification. Similar to the analysis of rheological properties, native potato starch stands out from the other starches.

### 2.4. Molecular Structure of Starch Preparations

The functional properties of starch in aqueous media depend both on the configuration of macromolecules and their conformation in solution. The method of choice for this type of study is size exclusion chromatography with triple detection. This method, thanks to the use of refractometric, viscometric and light scattering detectors, provides information on the molecular mass distribution, as well as several parameters describing the size and shape of the starch macromolecules in the solution [56,57,58]. Concerning the data presented in Table 4, it is worth noting that CS preparations soluble in cold water were characterized by significantly higher molecular mass values. In other words, hydrothermal treatment of starch, applied to obtain cold-water soluble preparations, was associated with an increase in the molecular mass of this polymer. This information may seem surprising as many years of experience of starch technologists and numerous literature reports prove the reduction of starch viscosity in the process of pregelatinization and drum-drying [38,39,40,59,60]. Despite that, the relationship between hydrothermal treatment and the increase in molecular weight while reducing the viscosity of pastes was presented [61]. The explanation of the observed phenomena is possible by the analysis of the derivative values calculated based on the comparison of data from the three detectors. First of all, the increase in the molecular mass of CS starch is associated with an increase in the degree of branching. Branched starches adopt such a conformation in the solution that their hydrodynamic radius does not change significantly. The radius of gyration changes slightly more, which results in changes in the Rg/Rh ratio, which is a measure of the shape of macromolecules in solution. It is assumed that compact spheres are described by the Rg/Rh value of 0.778, while coils and rods have a value > 2.0 [62]. Also, the lower than 0.5 values of the Mark–Houwink α coefficient, observed for all analysed samples, indicates the branched structure of macromolecules [63].

Chemical modification caused significant changes in the molecular mass of starch Table 4). As expected, an increase in the number and weight of the average molecular masses was observed due to the starch cross-linking process. It may seem surprising that the z-average molecular weight of chemically unmodified starch was the highest. An explanation for this paradox is possible if is considered that each of these parameters is related to different physical quantities. The number-average molecular mass is directly defined by the number of starch macromolecules, while the weight-average molecular mass is determined by the weight fractions of individual fractions with the same molecular mass. As a consequence, the M_n_ value is most defined by small molecules, while the M_w_ value is most determined by larger ones. The z-average molecular mass is related to light scattering processes in colloidal starch dispersions and, therefore, favours the effect of the largest molecules in the sample. Lower M_z_ values of cross-linked starches accompanied by increases of M_n_ and M_w_ indicated that the cross-linking process preferentially took place with the participation of smaller starch macromolecules. This hypothesis is confirmed by the lower polydispersity values (M_w_/M_n_) of cross-linked starches than those observed in chemically unmodified starch. Dual modified starches E1414 and E1422 were generally characterised by lower molecular masses than distarch phosphate E1412; however, in our previous work, we found only a small effect of the degree of acetylation process on the molecular structure of this polysaccharide [64].

**Figure 4:**
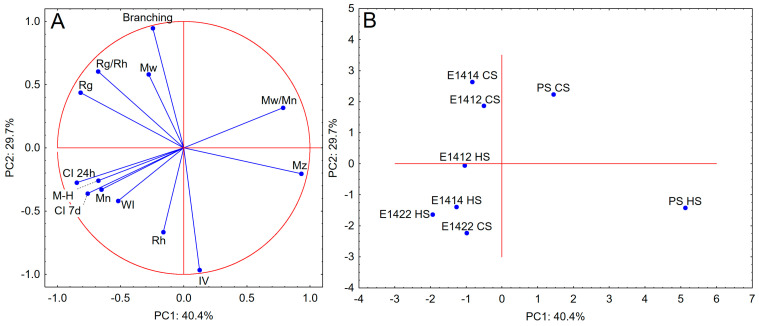
PCA analysis—(**A**) (loadings plot) and (**B**) (score plot) for parameters of stability of the emulsions and the parameters of proton relaxation phenomena (CI 24 h—creaming index after 24 h of storage; CI 7 d—creaming index after 7 days of storage; WI—whiteness index; M_n_—number average molecular mass; M_w_—weight average molecular mass; M_z_—z average molecular mass; M_n_/M_w_—polydispersity index; R_g_—radius of gyration; R_h_—hydrodynamic radius; IV—intrinsic viscosity; M-H—Mark–Houwink equation α parameter; Branching—number of branches per molecule.

PCA analysis (Figure 4) proved the correlation between emulsion stability parameters and some parameters of the size exclusion chromatography results. A strong positive correlation was found for the number average molecular mass, Mark–Houwink equation α parameter and stability parameters. The samples that represent this profile also show low M_w_/M_n_ values (E1414 HS, E1422 HS and E1422 CS). It might seem that interactions between solvent–polymer and polymer–polymer are crucial here since the higher the M–H α then the more a polymer molecule attracts the surrounding solvent than the mutual attraction of the molecules. However, the highly stable emulsion sample E1414 CS was positioned among the PS CS and E1414 CS samples. These samples show high M_w_, number of branches, R_g_/R_h_ and R_g_ values. The position of the PS HS sample is determined by a high M_z_. The E1412 HS sample is not well represented in this principal component system, being close to the origin of coordinates. The above shows that it is not possible to clearly indicate the starch parameters that are responsible for the stability of the tested emulsions. It seems that that the type of starch modification used is crucial here.

### 2.5. Surface Activity of Starch Preparations

**Figure 5:**
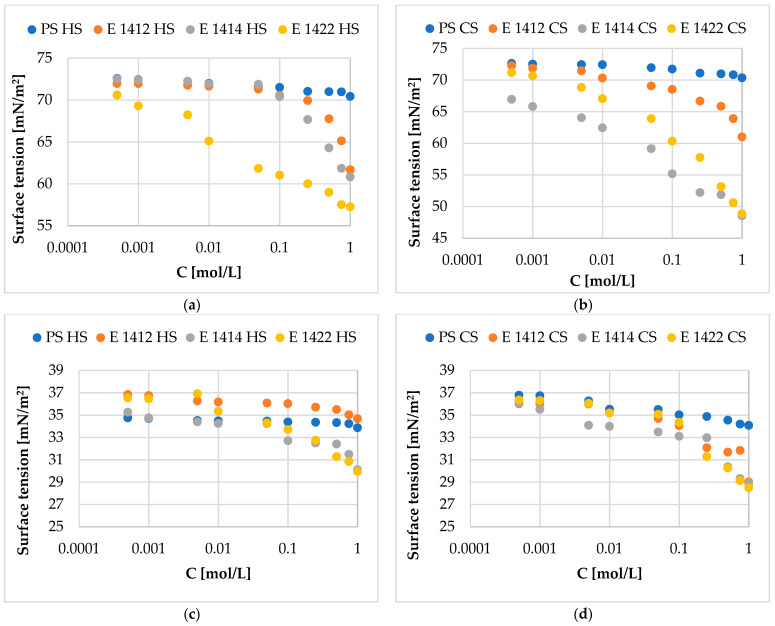
Surface tension (air/water system) (**a**,**b**) and interfacial tension (toluene/water system) (**c**,**d**) of the investigated starches: PS HS—native potato starch; PS CS pregelatinized potato starch; E1412 HS—distarch phosphate; E1412 CS—pregelatinized distarch phosphate; E1414 HS acetylated distarch phosphate; E1414 CS—pregelatinized acetylated distarch phosphate; E1422HS acetylated distarch adipate; E1414 CS—pregelatinized acetylated distarch adipate.

Surface and interfacial tension γ isotherms (Figure 5) showed that modification significantly changed the surface activity of starch. The surface tension values of the water/air system were lower in the presence of chemically modified starches than in the presence of native starch. Physical modification alone did not have any effect on surface tension. The strongest increase in surface activity was observed for cross-linked and additionally acetylated starches. In this case, physical modification caused a further decrease in the surface tension value. Similar, although less spectacular, results were obtained for the experiment at the water/toluene interface. Chemical modification of potato starch (irrespective of the type of modification) resulted in products that showed greater ability to reduce interfacial tension at the water/toluene interface compared to native starch. Also, in this case, it was observed that the physical modification of potato starch alone did not cause significant changes in the efficiency of reducing interfacial tension. However, dual physical and chemical modification resulted in higher interfacial activity of products than chemical modification alone. Similar observations were reported in the literature [65,66].

The values of the adsorption parameters resulting from the approximation of surface/interfacial tension isotherms data by the Szyszkowski equation are presented in Table 5 and Table 6. Their comparative analysis for native starch and chemically and physically modified derivatives allows for assessing the impact of chemical and physical modifications on the surface properties of starch preparations. In general, it should be noted that both chemical and physical modifications of starch reduce the tendency of starch preparations to adsorb. However, the type of modification has a significant impact on the packing and orientation of polysaccharide molecules in the saturated adsorption layer. In the relevant literature, there are very few reports that directly allow for the quantitative assessment of the effect of the starch modification process on its adsorption properties [67,68,69]. Only some of them concern the effect of modification (both physical and chemical) on the change in the functional properties of starch preparations, which are the effect of changes in adsorption properties. The studies were conducted for potato starch, but also for other botanical origins [70]. The value of ΔG_ads_ (Table 5) indicates that native potato starch molecules (potato HS) exhibit a strong tendency to adsorb at the water/air interface, but due to the size of the molecules, they form a loosely packed adsorption layer. As was mentioned above, the physical modification of the starch does not cause significant changes in the adsorption properties of the pregelatinized preparation PS CS. The ΔG_ads_ and Γ^∞^ values for native and gelatinized starch do not differ significantly. However, cross-linking of native starch results in a drastic reduction in the material’s tendency to adsorb at the interphase. The value of free energy of adsorption for the E1412 HS derivative is almost 10 times lower compared to ΔG_ads_ for native starch. Furthermore, cross-linking of the starch molecule is accompanied by a significant (more than 3-fold) reduction in the surface concentration of the adsorption layer formed at the water/air interface. Additional pregelatinization of the cross-linked starch preparation results in a significant increase in both the tendency to adsorb and the surface concentration at the saturated interphase. It is worth noting that the double-esterified derivative (E1414 HS) forms a similarly packed monolayer as the E1412 HS preparation. However, the pregelatinization of acetylated distarch phosphate, which increases the branching of polysaccharide macromolecules, results in a marked increase in both their tendency to adsorb at the water/air interface and the area occupied by statistical macromolecules in the saturated adsorption monolayer. On the other hand, the cross-linking process of esterified starch (E1422 HS), which increases the molecular mass and leads to the formation of bridges between macromolecules, results in a starch preparation that forms a highly densely packed monolayer of macromolecules in the water/air interface. In general, the increase in branching within starch macromolecules caused by cross-linking the starch preparation leads to changes in both the polysaccharide’s adsorption tendency and the packing density of the formed Gibbs adsorption layer. Pregelatinization of acetylated and cross-linked starch preparations further results in the compaction of molecules, which is reflected in the values of Γ^∞^ and A_min_. A similar trend can be observed in the water/toluene system. However, the changes in the values of individual adsorption parameters are not as significant.

**Figure 6:**
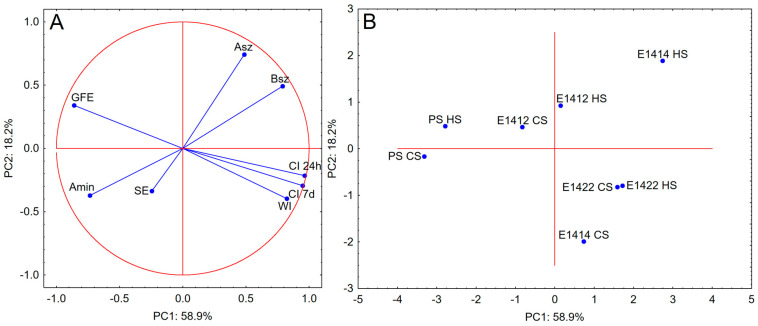
PCA analysis—(**A**) (loadings plot) and (**B**) (score plot) for parameters of the stability of the emulsions and the parameters of the surface activity of starches (CI 24 h—creaming index after 24 h of storage; CI 7 d—creaming index after 7 days of storage; WI—whiteness index; GFE—Gibbs free energy of adsorption (ΔG_ads)_; SE—Surface excess (Γ_∞_); A_min_—Surface area per macromolecule in the saturated adsorption monolayer; A_sz_—Szyszkowski isotherm coefficient; B_sz_—Szyszkowski isotherm coefficient.

The PCA analysis results showed a strong correlation between the Gibbs free energy of adsorption and the emulsion stability parameters. This indicates the crucial importance of thermodynamics in the formation and stabilization of emulsions. The other parameters of the Szyszkowski equation did not reveal any significant significance. The data presented in Figure 6B indicate that the presence of acetyl groups in the structure of starch macromolecules was of crucial importance for the surface activity of starch. Physical modification was also important as the PS CS and E1412 CS starches are located close together on the score plot. The preparations that differed the most were PS HS and E1412 HS. This means that cross-linking significantly changed the functionality of starch concerning the surface activity of potato starch.

## 3. Materials and Methods

### 3.1. Materials

Commercial food-grade chemically modified starches kindly provided by the potato processing company “Zetpezet” Ltd. (Piła, Poland) were the basic working material:

- Distarch phosphate E1412;
- Acetylated distarch phosphate E1414;
- Acetylated distarch adipate E1422.

All of the above starch variants were provided in hot and cold soluble (pregelatinized) variants. Native potato starch was used as a reference material. All samples were manufactured in the year 2018 and were produced from starch obtained during one production campaign.

### 3.2. Emulsion Preparation

The model emulsions were prepared with food-grade rapeseed oil and deionized water in a 1:3 ratio. A Silent Crusher homogenizer, Heidolph (Schwabach, Germany), was used to prepare the emulsion. Homogenization of the mixture was carried out for 3 min at 21,000 revolutions per second.

Emulsions using hot soluble starches (HS) were prepared based on 10% starch pastes that were gelatinized in a boiling water bath for 20 min. Oil was added to the cooled gelatinized starch pastes and afterwards, homogenization was performed to obtain an emulsion.

Emulsions from cold-soluble starch (CS) preparations were prepared similarly as is practised in the food industry for the preparation of sauces. To this end, 10% starch (*w*/*w* of water) was suspended in the oil, and subsequently, water was added and homogenization was performed to obtain an emulsion.

### 3.3. Methods

#### 3.3.1. Creaming Index

The creaming index was determined according to the procedure of Titus and co-authors modified by Acton and Saffle [71]. Emulsion samples were placed in centrifuge tubes and incubated at 37 °C for 24 h and 7 days. Subsequently, the samples were centrifuged at 2500× *g* for 10 min. The creaming index (CI) was calculated from the following formula:

CI = (V_m_ − V_o_)/V_m_ • 100%, (1)

where: V_m_—the initial volume of the emulsion [cm^3^] and V_o_—the volume of the separated oil [cm^3^].

#### 3.3.2. Colour Measurements

Colour measurements of the emulsions were made using the Minolta Chroma Meter CR-310 (Japan) colourimeter. Measurement conditions were chosen: observer 2°, illuminant C, colour space CIE L*a*b*. Absolute colour parameters as well as the whiteness index (WI) were determined:

WI = 100 − √((100 − L)^2^ + a^2^ + b^2^) (2)

where: L*—lightness, a value between 0 (black) and 100 (white); a—chromatic coordinate indicating red (positive values) and green (negative values) balance; and b—chromatic coordinate indicating yellow (positive values) and blue (negative values) balance.

#### 3.3.3. Rotational Rheometry

The rheological properties of the emulsions were determined using a RotoVisco1 rheometer (Haake Technik GmbH, Vreden, Germany) equipped with a DC30-K10 refrigerated bath with an immersion circulator. Before the measurement, the samples were brought to 20 °C and relaxed in a measuring cylinder for 5 min. Data collection and calculations were made using RheoWin 3.61 software. The following measurements were performed:

Flow curves were determined using the Z20 DIN measurement system, within 1–600–1 s^−1^ shear rate in a time of 2 min. Obtained flow curves were described with the Ostwald de Waele equation.

Temperature versus apparent viscosity curves were determined at 100 s^−1^ shear rate in the 20 to 65 °C temperature range and a heating speed of 3 °C/min. Samples were pre-sheared at 100 s^−1^ for 180 s. The obtained curves were described by the Arrhenius Temperature–Time Superposition equation.

#### 3.3.4. Low Field NMR

NMR relaxation times were analysed with a pulse NMR spectrometer PS15T operating at 15 MHz (Ellab, Poznań, Poland) in triplicate. The inversion–recovery (180-TI-90) pulse sequence was applied for measurements of the T_1_ relaxation times. The 180 pulse was 4.8 μs, the distance between RF pulses (TI) changed from 2 to 1800 ms, and the repetition time TR between sequences was 18 s.

Measurements of the spin-spin (T_2_) relaxation times were taken using the pulse train of the Carr–Purcell–Meiboom–Gill spin echoes (90-TE/2-(180)_n_). The echo time TE was 3 ms, and the number of the spin-echoes signal (n) was 50. The five accumulations of the spin-echo trains were used. The repetition time between pulse trains was 15 s.

#### 3.3.5. Size Exclusion Chromatography

Starch samples were dissolved in DMSO at 80 °C for 4 days with gentle stirring (Reacti-Therm, Thermo Fisher Scientific, Waltham, MA, USA). The concentration of the sample separated with SEC was 1 mg/mL. The samples were not filtered prior to analysis. The injection volume was 100 μL. SEC equipment with triple detection (Viscotek 305 TDA) was used (Malvern Practical, Malvern, UK). A conventional dual cell refractometer, viscometer, and light scattering detectors (low-angle light scattering, LALS and right-angle light scattering RALS) were employed to act together. One organic SEC column (PSS GRAM series, 3000 Å, 8 × 300 mm) with a guard column (Polymer Standard Service GmbH, Mainz, Germany) was applied. The flow rate of DMSO was 0.3 mL/min at 70 °C. The calibration was performed with the pullulan standard (113,000 g/mol). The refractive index (RI) of the solvent was 1.4595 [72]. The calculations were performed using OmiSEC 4.7 software (Malvern, TX, USA) assuming the increment in the refractive index (dn/dc) for starch in DMSO is 0.0659 mL/g. The Zimm–Stockmayer equation for a randomly branched polydisperse polymer was used to calculate the number of branches per molecule (Bn). Due to the lack of an appropriate linear standard for starch, the pullulan standard was used as a linear molecule. The determined M–H (a) = 0.710 and the M–H intercept (log K = −3.175) for pullulan in DMSO were used. The parameters obtained from the SEC separations were the mean of three independent trials.

#### 3.3.6. Surface Properties

The surface-active properties of all starches obtained were studied by the equilibrium and dynamic surface/interfacial tension experiments performed in two systems, namely air/water and oil/water.

The equilibrium surface/interfacial tension of the starch samples was measured by the du Noüy ring method with a KRÜSS K12 tensiometer (Hamburg, Germany) [73]. The standard deviation of the surface/interfacial tension measurements was 0.05 mN/m. All measurements were carried out at 21 ± 0.1 °C and repeated three times. Twice distilled water with a conductivity of 3 μS was used as the aqueous phase. The toluene used as the organic phase for interfacial tension measurements was obtained from Aldrich Company (St. Louis, MS, USA). It is obvious that toluene cannot be used as a food additive; however, it was applied in our investigations as the model organic phase [30].

The experimentally determined surface/interfacial tension isotherms were approximated using the Szyszkowski equation. Based on the coefficients of the Szyszkowski equation, the values of adsorption parameters were estimated, such as the Gibbs free energy of adsorption, surface excess at the saturated interphase, and the area occupied by a statistical molecule adsorbed at the saturated interphase.

#### 3.3.7. Statistical Analysis

All analyses were performed in triplicate (unless otherwise stated), and the results are presented as mean value ± standard deviation. Principal component analysis (PCA) was performed based on the correlation matrix. The statistical analyses were performed using Statistica 13.3 (TIBCO Software Inc., Palo Alto, CA, USA).

## 4. Conclusions

The studies presented above have shown that the stability of an emulsion is influenced by several factors but their significance varies considerably. Although the primary factor determining the functionality of starch preparations is their molecular structure, the proton relaxation phenomena occurring in the continuous phase of the emulsion, which are its consequences, turned out to be crucial for the stability of the emulsion. The thermodynamics of the adsorption of starch macromolecules at the water/oil interface was also of great importance.

Modification significantly improves the applicability of starch for emulsion stabilization. This applies not only to cross-linking and acetylation but also to physical modification. This is because not only cross-linking but also pregelatinization causes changes in the molecular structure of starch, including an increase in the molecular weight and the degree of branching of macromolecules. This implies changes in the conformation of the starch macromolecules in solution and in proton relaxation processes affects their ability to adsorb in the water/oil interface and, consequently, in their ability to stabilize the emulsion.

## Figures and Tables

**Table 1:** Instrumentally determined colour parameters of model emulsions with different starches in the CIE L*a*b* system.

	L (Whiteness)	a (Red–Green Balance)	b (Yellow–Blue Balance)	WI (%)
PS HS	66.85	−1.89	0.54	72.7
PS CS	67.04	−1.97	0.42	67.0
E1412 HS	69.59	−2.48	1.94	69.4
E1412 CS	72.71	−2.46	1.30	72.6
E1414 HS	83.85	−2.88	2.43	83.4
E1414 CS	85.07	−2.87	1.41	84.7
E1422 HS	86.74	−3.19	2.36	86.2
E1422 CS	85.52	−3.13	1.83	85.1

**Table 2:** Rheological parameters of the emulsions.

	Consistency Index K [Pa·s^n^]		Flow Behaviour Index n [−]		Thixotropy [Pa·s^−1^]		Flow Activation Energy E [J·mol^−1^]	
	HS	CS	HS	CS	HS	CS	HS	CS
PS	1.37	4.32	0.8504	0.6824	19,480	57,465	216.1	230.1
E1412	32.48	4.05	0.3579	0.7108	−29,212	63,705	162.7	245.0
E1414	21.62	10.81	0.5593	0.6572	23,260	5960	278.3	293.1
E1422	11.66	3.48	0.6342	0.7974	15,365	−18,305	257.3	274.8

**Table 3:** The data of proton relaxation phenomena in the emulsions studied.

	Short Component of the Spin-Lattice Relaxation Time T_11_		Short Component of the Spin-Spin Relaxation Time T_21_		Long Component of the Spin-Lattice Relaxation Time T_12_		Long Component of the Spin-Spin Relaxation Time T_22_	
	2 h	24 h	2 h	24 h	2 h	24 h	2 h	24 h
PS HS	57.8 ± 0.9	79.2 ± 0.7	54.5 ± 3.7	69.9 ± 5.6	805.7 ± 6.1	829.2 ± 2.7	502.5 ± 6.2	451.1 ± 6.8
PS CS	60.9 ± 0.9	75.2 ± 2.0	39.2 ± 8.5	44.8 ± 5.2	841.8 ± 4.3	847.1 ± 1.1	460.5 ± 1.7	466.8 ± 5.9
E1412 HS	67.5 ± 0.9	64.1 ± 0.7	55.9 ± 3.8	39.3 ± 2.9	858.1 ± 6.0	840.1 ± 5.0	455.5 ± 6.1	394.2 ± 4.3
E1412 CS	71.5 ± 1.2	68.1 ± 2.1	57.9 ± 4.1	54.5 ± 2.2	827.7 ± 4.7	820.1 ± 3.1	395.1 ± 4.2	394.8 ± 1.5
E1414 HS	74.3 ± 0.6	69.1 ± 0.7	66.3 ± 4.9	58.9 ± 4.2	884.8 ± 2.9	829.2 ± 2.7	532.4 ± 5.8	522.1 ± 6.8
E1414 CS	66.7 ± 1.2	65.5 ± 1.2	53.6 ± 8.4	50.3 ± 1.5	850.5 ± 5.9	858.8 ± 9.9	510.5 ± 1.7	559.1 ± 2.7
E1422 HS	67.0 ± 0.5	65.8 ± 0.5	47.9 ± 8.0	490. ± 3.3	881.6 ± 3.6	862.4 ± 2.2	525.7 ± 2.0	536.3 ± 4.2
E1422 CS	670. ± 0.8	65.3 ± 1.2	49.9 ± 2.7	49.7 ± 3.4	866.5 ± 3.6	855.4 ± 3.3	559.9 ± 6.7	561.5 ± 4.4

**Table 4:** Molecular masses and hydrodynamic parameters of investigated starches.

	Potato Starch		E1412		E1414		E1422	
Parameter	HS	CS	HS	CS	HS	CS	HS	CS
M_n_ [Dalton]	2.52 × 10^6^ ± 3.61 × 10^5^	6.46 × 10^6^ ± 5.04 × 10^5^	1.74 × 10^7^ ± 2.25 × 10^6^	1.08 × 10^7^ ± 1.31 × 10^6^	7.74 × 10^6^ ± 1.55 × 10^5^	3.68 × 10^6^ ± 1.68 × 10^5^	1.52 × 10^7^ ± 4.53 × 10^6^	1.27 × 10^7^ ± 1.53 × 10^6^
M_w_ [Dalton]	2.87 × 10^7^ ± 7.71 × 10^5^	4.20 × 10^7^ ± 1.23 × 10^6^	4.22 × 10^7^ ± 1.36 × 10^6^	4.57 × 10^7^ ± 1.07 × 10^6^	3.24 × 10^7^ ± 1.20 × 10^5^	3.48 × 10^7^ ± 1.01 × 10^6^	3.14 × 10^7^ ± 2.23 × 10^6^	3.72 × 10^7^ ± 9.12 × 10^5^
M_z_ [Dalton]	6.10 × 10^8^ ± 6.43 × 10^6^	9.99 × 10^7^ ± 4.36 × 10^6^	9.18 × 10^7^ ± 2.29 × 10^7^	9.59 × 10^7^ ± 7.93 × 10^6^	6.13 × 10^7^ ± 1.46 × 10^6^	9.27 × 10^7^ ± 8.94 × 10^6^	5.15 × 10^7^ ± 4.44 × 10^6^	6.16 × 10^7^ ± 5.62 × 10^6^
M_w_/M_n_	11.52 ± 1.95 ^ab^	6.51 ± 0.32 ^ab^	2.46 ± 0.40 ^ab^	4.28 ± 0.62 ^ab^	4.19 ± 0.07 ^ab^	9.48 ± 0.71 ^ab^	2.14 ± 0.49 ^a^	2.95 ± 0.43 ^ab^
Intrinsic viscosity [IV] [dl/g]	1.407 ± 0.044 ^c^	0.935 ± 0.097 ^ab^	1.130 ± 0.219 ^abc^	1.068 ± 0.052 ^abc^	1.372 ± 0.007 ^c^	0.825 ± 0.055 ^a^	1.343 ± 0.057 ^c^	1.385 ± 0.038 ^c^
Hydrodynamic radius R_h_—[nm]	79 ± 2 ^c^	77 ± 4 ^bc^	85 ± 2 ^cd^	82 ± 0 ^cd^	80 ± 0 ^cd^	66 ± 0 ^ab^	83 ± 1 ^cd^	87 ± 1 ^d^
Radius of gyration R_g_—[nm]	96 ± 6 ^b^	166 ± 2 ^cde^	187 ± 0 ^cde^	227 ± 10 ^f^	197 ± 9 ^cdef^	214 ± 22 ^ef^	212 ± 15 ^ef^	149 ± 5 ^cd^
R_g_/R_h_	1.22 ± 0.10 ^ab^	2.15 ± 0.13 ^bcde^	2.19 ± 0.06 ^e^	2.76 ± 0.12 ^abcd^	2.46 ± 0.11 ^e^	3.24 ± 0.30 ^abcde^	2.56 ± 0.15 ^cde^	1.72 ± 0.08 ^de^
Mark–Houwink α	0.279 ± 0.073 ^ab^	0.376 ± 0.004 ^bcde^	0.459 ± 0.010 ^e^	0.305 ± 0.011 ^abcd^	0.446 ± 0.001 ^e^	0.362 ± 0.016 ^abcde^	0.389 ± 0.016 ^cde^	0.406 ± 0.037 ^de^
Number of branching	1010 ± 56 ^b^	3387 ± 145 ^c^	2481 ± 347 ^e^	3322 ± 4 ^bc^	1488 ± 68 ^e^	3558 ± 171 ^c^	1629 ± 186 ^d^	1736 ± 155 ^e^

Explanatory notes: values marked with the same letter do not differ significantly *p* > 0.05.

**Table 5:** Adsorption parameters at the water/air interface of the investigated starches.

Starch	Gibbs Free Energy of Adsorption –ΔG_ads_ [kJ/mol]	Surface Excess Γ^∞^ × 10^7^ [mol/m^2^]	Surface Area Per Macromolecule in the Saturated Adsorption Monolayer A_min_ × 10^18^ [nm^2^]	Szyszkowski Isotherm Coefficient A_sz_ [mol/dm^3^]	Szyszkowski Isotherm Coefficient B_sz_ [–]
potato HS	31.37	1.03	16.14	0.000003	0.0034
potato CS	28.02	1.14	14.52	0.000012	0.0038
E1412 HS	3.50	0.27	0.61	0.243	0.091
E1412 CS	15.93	6.57	2.53	0.0016	0.022
E1414 HS	5.46	0.23	0.72	0.11	0.078
E1414 CS	24.15	0.10	1.66	0.00005	0.034
E1422 HS	22.85	7.35	2.26	0.0001	0.025
E1422 CS	14.75	0.16	1.04	0.0026	0.054

**Table 6:** Adsorption parameters at the water/toluene interface of the investigated starches.

Starch	Gibbs Free Energy of Adsorption –ΔG_ads_ [kJ/mol]	Surface Excess Γ^∞^ × 10^7^ [mol/m^2^]	Surface Area Per Macromolecule in the Saturated Adsorption Monolayer A_min_ × 10^18^ [nm^2^]	Szyszkowski Isotherm Coefficient A_sz_ [mol/dm^3^]	Szyszkowski Isotherm Coefficient B_sz_ [−]
Potato HS	26.44	32.08	51.76	0.00002	0.0023
Potato CS	18.40	1.53	10.87	0.0006	0.01
E1412 HS	17.72	1.07	15.55	0.0008	0.0072
E1412 CS	12.13	5.40	3.08	0.0074	0.036
E1414 HS	24.97	2.22	7.49	0.00004	0.015
E1414 CS	18.95	3.70	4.49	0.0005	0.025
E1422 HS	11.46	5.80	2.86	0.0097	0.039
E1422 CS	8.32	0.10	1.64	0.035	0.068

## Data Availability

The original contributions presented in this study are included in the article; further inquiries can be directed to the corresponding author.
